# Characterisation and management of therapy-interrupting system errors associated with the use of the HomeChoice cycler for automated peritoneal dialysis

**DOI:** 10.1186/s12913-026-15289-1

**Published:** 2026-08-08

**Authors:** Annemarie Albert, Stefan Richter, Rainer Peter Woitas, Philipp Stieger, Rüdiger C. Braun-Dullaeus, Christian Albert

**Affiliations:** 1 Diaverum Renal Services, Am Neuen Garten 11, 14469 Potsdam, Germany; 2 Department of Nephrology, Central Clinic Bad Berka, Robert‑Koch‑Allee 9, 99438 Bad Berka, Germany; 3 Diaverum Deutschland GmbH, Hansastraße 23, 80686 München, Deutschland; 4 University Clinic for Cardiology and Angiology, Otto‑von‑Guericke University Magdeburg, Leipziger Str.44, 39120 Magdeburg, Germany

**Keywords:** Peritoneal dialysis, Home therapy, Renal replacement therapy, Telemedicine, Nursing, Automated peritoneal dialysis, APD, HomeChoice, Cycler

## Abstract

**Background:**

Methodological and technical education of nurses is required to address challenges of technology integration in peritoneal dialysis (PD). We analysed the nature of therapy interrupting system errors in automated peritoneal dialysis (APD) cyclers of a German PD reference care centre and provide applicable resolution proposals.

**Methods:**

We documented therapy interrupting system errors of HomeChoice Pro and HomeChoice Claria cyclers (Baxter, BX, Unterschleißheim, Germany) including the years 2015-2023. We systematically characterise and suggest resolution strategies to these errors based on routinely collected health data and user experience to safely guide nephrology professionals and patients during troubleshooting. The ‘reporting of studies conducted using observational routinely collected health data’ (RECORD)-statement was considered.

**Results:**

We identified 49 different therapy interrupting system errors which were assigned to eight major categories: Errors associated to the pressure chamber (N=18), the Digitalboard/EEPROM (N=18, Electrically Erasable Programmable Read Only Memory), air in set (N=3), opening of the set’s door (N=3), those associated to the energy system (N=3), the heater system (N=3) and a variety of errors resulting in an abrupt termination or non-commencement of the treatment session without clear group assignment (undefined, N=1) or without sufficient documentation. In total, 138 error incidents were documented during the observational timeframe. In frequential order errors were attributed to the pressure chamber (21.74%,), and air in set (19.57%) where specifically error 2240 occurred in 17.39% of total cases, the Digitalboard/EEPROM (17.39%), the energy or battery (16.67%; No. 1032 in 19/23 cases), the set’s door (7.25%) and the heater (4.35%). In 17 cases (12.32%) no error number was documented by the patient or staff. One error cause was registered as undefined (0.72%). Overall, errors were rarely occurring in <0.25% of treatment sessions performed in the observational timeframe.

**Conclusion:**

These findings may be useful to guide training purposes for nephrology personnel and persons doing APD. For the manufacturer, the information provided may be helpful to improve error management and information transmission to the persons doing APD with the HomeChoice devices.

## Background

Peritoneal dialysis (PD) is an increasingly provided renal replacement therapy for patients with end stage renal disease, enabling the provision of a home-based therapy [1, 2]. Automated peritoneal dialysis (APD) is a modality that is usually performed overnight and facilitates the implementation of automated fill, dwell and drain cycles tailored to patient’s needs [3]. Use of this therapeutic option has enabled an optimized management of ultrafiltration (UF) and achievement of improved dialytic clearance and outcome, specifically for persons with high peritoneal solute transfer status (attributed ‘high transporter’) [4].

APD cyclers have undergone significant technological improvements since their introduction in the 1960s [5]. The Homechoice Claria is one of the most recent cyclers that is connected to a cloud-based interface by a 5G-modem, and after finishing a therapy session, automatically uploads and provides associated data to the clinical care team for review [6]. Programming the dialysis prescription into the cycler’s software is usually carried out by the nursing staff, who are confronted with the challenge of progressive digitalization and associated need for technology integration in clinical nephrology.

HomeChoice cyclers are set up for use by a 5-button interface and a two-line display screen guiding the user through the treatment program. Modern cyclers are controlled by software programs running in the device’s background usually unnoticed by the person doing APD. They monitor the course of the treatment, pumps, heating, and flow of dialysis solutions and ensure patients’ safety in the progress. However, all those parts may be prone to errors.

In general, for the HomeChoice cyclers, system errors must be differentiated from alarms brought about by the software: Alarms prompt the user to react on a certain problem identified by the device and displayed on the screen. The user must resolve the problem and can subsequently continue with the therapy [7].

Genuine errors occurring in the system force an interruption of the program and the ongoing therapy and may represent relevant issues for patients and staff. Accordingly, appropriate methodological expertise is needed to address such challenges and to provide safe and effective therapy in the clinic or in homecare [8]. Moreover, limited information is available on therapy interrupting cycler system errors concerning persons doing APD and nurses in case of error incidents [7], and no previous study reported on the subject and management of such complications [9, 10].

Therefore, the aim of this study was to systematically categorize therapy interrupting system errors, and if technically possible, to provide applicable resolution proposals for save continuity of treatment.

## Materials and methods

### Data collection, categorization, and analysis

We prospectively documented all therapy interrupting system errors of the HomeChoice cyclers in use at our clinic and homecare including complete years 2015–2023. Standardised error protocols for documentation were filed for each patient- or staff reported system error. Access to sensitive clinical patient data was reserved for Diaverum affiliated authors and not shared with third parties. For the retrospective analysis of this routinely collected clinical patient record data three readers, two nephrologists (AA and CA) and the head nurse (SR) independently reviewed all protocols. The total and proportionate occurrence of each error was documented. Actions taken to successfully initiate, resume or finalize a treatment session are proposed for each system error. During the observational timeframe, we exchanged information with the manufacturer for validation and clarification of our findings, and each previously undocumented system error and those not featured in the operating instructions were reported.

Finally, the technical description and classification of system errors presented in the main results Tables 1 and 2 were proofread by Baxter Healthcare Germany. According to the study protocol, the extracted data was anonymized immediately upon access to the patients’ clinical record and no other than retrospective routine clinical data was collected or analysed.

**Table 1:** Categorization of individual therapy interrupting system errors and families

Error No.	Family	Category
3		Digitalboard
7		Digitalboard
106	1xx	Pressure chamber
151	149–155	Pressure chamber
153	149–155	Pressure chamber
185	176–181, 184–188	Pressure chamber
167	166–169	Pressure chamber
1031	103x	Energy or battery
1032	103x	Energy or battery
1034		Digitalboard
1036		Digitalboard
1087		Undefined
1102	110x	Heater
1103	110x	Heater
2040		Pressure chamber
2041	2041–2046	Pressure chamber
2042	2041–2046	Pressure chamber
2043	2041–2046	Pressure chamber
2044	2041–2046	Pressure chamber
2045	2041–2046	Pressure chamber
2046	2041–2046	Pressure chamber
2058	2052, 2057–2058	Heater
2065	2061–2083	Digitalboard
2066	2061–2083	Digitalboard
2067	2061–2083	Digitalboard
2068	2061–2083	Digitalboard
2069	2061–2083	Digitalboard
2070	2061–2083	Digitalboard
2071	2061–2083	Digitalboard
2083	2061–2083	Door opened
2084	208x?	Door opened
2096	1085–2096	Pressure chamber
2098		Pressure chamber
2206		Pressure chamber
2240	224x	Air in set
2245	2241–2254	Pressure chamber
2248	2241–2254	Pressure chamber
2265		Door opened
2267	226x–2267	Air in set
2284	226x–228x	Pressure chamber
2298	2294–3000	Digitalboard
2306	2304–2326	Digitalboard
2317	2304–2326	Digitalboard
2365	2334–2389	Digitalboard
2367	2334–2389	Air in set
3175		Digitalboard
3572		Digitalboard
4644		Digitalboard
batry		Energy or battery
nnn?		Undocumented

The pressure chamber is also called cassette chamber in certain publications; Air in set, detection of air in the disposable tubing set; Digitalboard, error associated to the Digitalboard or EEPROM, Electrically Erasable Programmable Read Only Memory; Door opened, associated to the pressure chamber door or hatch

**Table 2:** Characterization and resolution proposals for therapy interrupting system errors

Error No.	Description	Solution proposal
3	Software error, usually not recoverable (green button does not respond to input)	Switch off, restart and re-initialize program
7	Software errors, usually not recoverable	No resolution identified
106*	Loss of pressure in the system, usually not recoverable, (Dialysate flow to high)	No resolution identified, exchange Cycler
151*	Loss of pressure in the system, usually not recoverable (cannot build up pressure due to air leackage)	Close all clamps. Switch off, change set and re-initialise program. Eventually exchange Cycler
153*	Loss of pressure in the system, usually not recoverable (cannot build up pressure due to air leackage)	No resolution identified
185*	Loss of pressure in the system, usually not recoverable, (Pressure measurement)	Close all clamps. Switch off, change set and re-initialise program
167*	Internal pressure leaks, usually not recoverable, (air in the system)	No resolution identified; Cycler exchanged in most cases
1031	On/off switch does not contact properly, (Overload protection)	Disconnect the power supply and reinitialize program
1032	On/off switch does not contact properly, (Overload protection)	Disconnect the power supply and reinitialize program
1034	Signal digital board error, occurs very rarely, (Treatment is not initiated)	Close clamps. Disassemble set, disconnect power supply and reinitialize program
1036	Signal digital board error, occurs very rarely, (Cycler repeatedly dispays ‘SYSTEM ERROR 1036’)	No resolution identified, exchange Cycler
1087	Cycler repeatedly dispays ‘SYSTEM ERROR 1087’, repeated bypass performed?	Finished treatment without further issues
1102†	Heater does not work	Heater malfunction, disconnect power supply, reinitialize program, exchange cycler
1103†	Heater does not work	Heater malfunction, disconnect power supply, reinitialize program, exchange cycler
2040	High pressure loss bladder or tank defective (Pressure chamber)	Close all clamps. Switch off, change set and re-initialise program
2041	High pressure loss bladder or tank defective (Pressure chamber)	Close all clamps. Switch off, change set and re-initialise program
2042^§^	High pressure loss bladder or tank defective (Pressure chamber), check if cassette is properly inserted	Close all clamps. Switch off, change set and re-initialise program
2043	High pressure loss bladder or tank defective (Pressure chamber)	Close all clamps. Switch off, change set and re-initialise program
2044^§^	High pressure loss bladder or tank defective (Pressure chamber)	Close all clamps. Switch off, change set and re-initialise program
2045	High pressure loss bladder or tank defective (Pressure chamber)	Close all clamps. Switch off, change set and re-initialise program
2046^§^	High pressure loss bladder or tank defective (Pressure chamber)	Close all clamps. Switch off, change set and re-initialise program
2058	Heater temperature below 31 degrees celcius. Occured after Softwareupdate.	Switch off and on, re-initialize program to reactivate heater
2065†	Software error, switch off/on, not always recoverable	Close all clamps. Press the power switch Off and On to end the therapy.^#^
2066†	Software error, switch off/on, not always recoverable	Close all clamps. Press the power switch Off and On to end the therapy.^#^
2067†	Software error, switch off/on, not always recoverable	Close all clamps. Press the power switch Off and On to end the therapy.^#^
2068†	Software error, switch off/on, not always recoverable	Close all clamps. Press the power switch Off and On to end the therapy.^#^
2069†	Software error, switch off/on, not always recoverable	Close all clamps. Press the power switch Off and On to end the therapy.^#^
2070†	Software error, switch off/on, not always recoverable	Close all clamps. Press the power switch Off and On to end the therapy.^#^
2071†	Software error, switch off/on, not always recoverable	Close all clamps. Press the power switch Off and On to end the therapy.^#^
2083	Illegal door opening or door handle broken, magnet or sensor or user	Vent if option given. Otherwise, close clamps. Switch off, replace set and re-initialise program
2084	PD set associated error; air in system; follow up error of 2265; door opened during use, magnet or sensor or user,	Vent if option given. Otherwise, close clamps. Switch off, replace set and re-initialise program
2096	Error associated to the pressure chamber; follow up error of 2041–2045 (negative tank leaking)	Vent if option given. Otherwise, close clamps. Switch off, replace set and re-initialise program
2098*†	usually internal pressure loss, if specific error occurred more than 4 times	Close all clamps. Press the power switch Off and On to end the therapy.^#^
2206	Cycler stopped treatment during cycle 4 of 6 (pressure test failed)	Supply bag punctured? Check if enough solution for flushing of lines is available. Drain manually.
2240	Air in the tubing set exceeds more than 3mL, no device error	Vent if option given. Otherwise, close clamps. Switch off, replace set and re-initialise program
2245	Failed pressure testing, usually internal pressure loss	Vent if option given. Otherwise, close clamps. Switch off, replace set and re-initialise program
2248	Failed pressure testing, usually internal pressure loss	Vent if option given. Otherwise, close clamps. Switch off, replace set and re-initialise program
2265	Illegal door opening or door handle broken, magnet or sensor or user, (Follow up error associated to 2084 or 2412)	Close clamps, disassemble set, disconnect power supply and reinitialize program
2267	Air in the tubing set exceeds more than 3ML, no device error; The system detected air in the disposable tubing set and solution bag and diverted flow to drain. Follow up error, frequently associated to 2240.	Vent if option given. Otherwise, close clamps. Switch off, replace set and re-initialise program
2284	Failed the pressure testing; Error associated to the set; Follow up error of 2265	Close clamps. Disassemble set, disconnect power supply and reinitialize program
2298	Digitalboard and software error	No resolution identified
2306	Digitalboard and software error	Close clamps, disassemble set, drain manually. Check if enough solution is available/accessible.
2317	Digitalboard and software error (treatment is not initiated)	Close clamps, change set and/or re-initialise program
2365	Software error, switch off/on, not always recoverable	No resolution, drain manually
2367	Software error, switch off/on, not always recoverable, associated to Air in Set, follow up error, frequently associated to 2240 and 2267 after switching cycler Off and On, all clamps set correctly?	Vent if option given. Otherwise, close clamps. Switch off, replace set and re-initialise program
3175	Digitalboard, software error, EEPROM	No resolution identified
3572	Digitalboard, software error, EEPROM	No resolution identified
4644	Digitalboard, software error, EEPROM	No resolution identified, exchange Cycler
batry	BATTERY ERRROR (BATTERIE FEHLER)	Exchange Cycler
nnn?	SYSTEM ERROR nnn?	System error number not documented.

Legend: * These errors were usually associated to internal part malfunction that needed service replacements. Air in set, detection of air in the disposable tubing set; ^†^also documented in HomeChoice Pro user guide; ^§^ If this alarm occurs before the patients' connection it is to be treat as a 'Reload Set Alarm'. ^#^ Recommendation given in the cycler manual

Abbreviations: Cassette, The clear rectangular plastic piece of the disposable set that is inserted behind the door of the HomeChoice cycler housing the pressure/pump system; The pressure chamber is also called cassette chamber in certain publications; EEPROM, Electrically Erasable Programmable Read Only Memory; For safety reasons, lines and bags should also be changed when changing sets to prevent contamination

### Materials and software

Current HomeChoice Claria cyclers with Sharesource remote connectivity (Baxter, BX, Unterschleißheim, Germany) were used for home-based treatment and intermittent PD treatment in the clinic (IPD). The HomeChoice Pro cyclers are the previous generation cyclers that were used as backup machines. Physioneal, icodextrin and nutrineal solutions (BX) were prescribed by the treating nephrologists independent of this investigation.

The ‘reporting of studies conducted using observational routinely collected health data’ (RECORD)-statement was acknowledged [11]. All statistical analyses were performed using the R environment for statistical computing (R Foundation for Statistical Computing, Vienna, Austria) [12].

## Results

### Categorization of individual therapy interrupting system errors and families

**Fig. 1:**
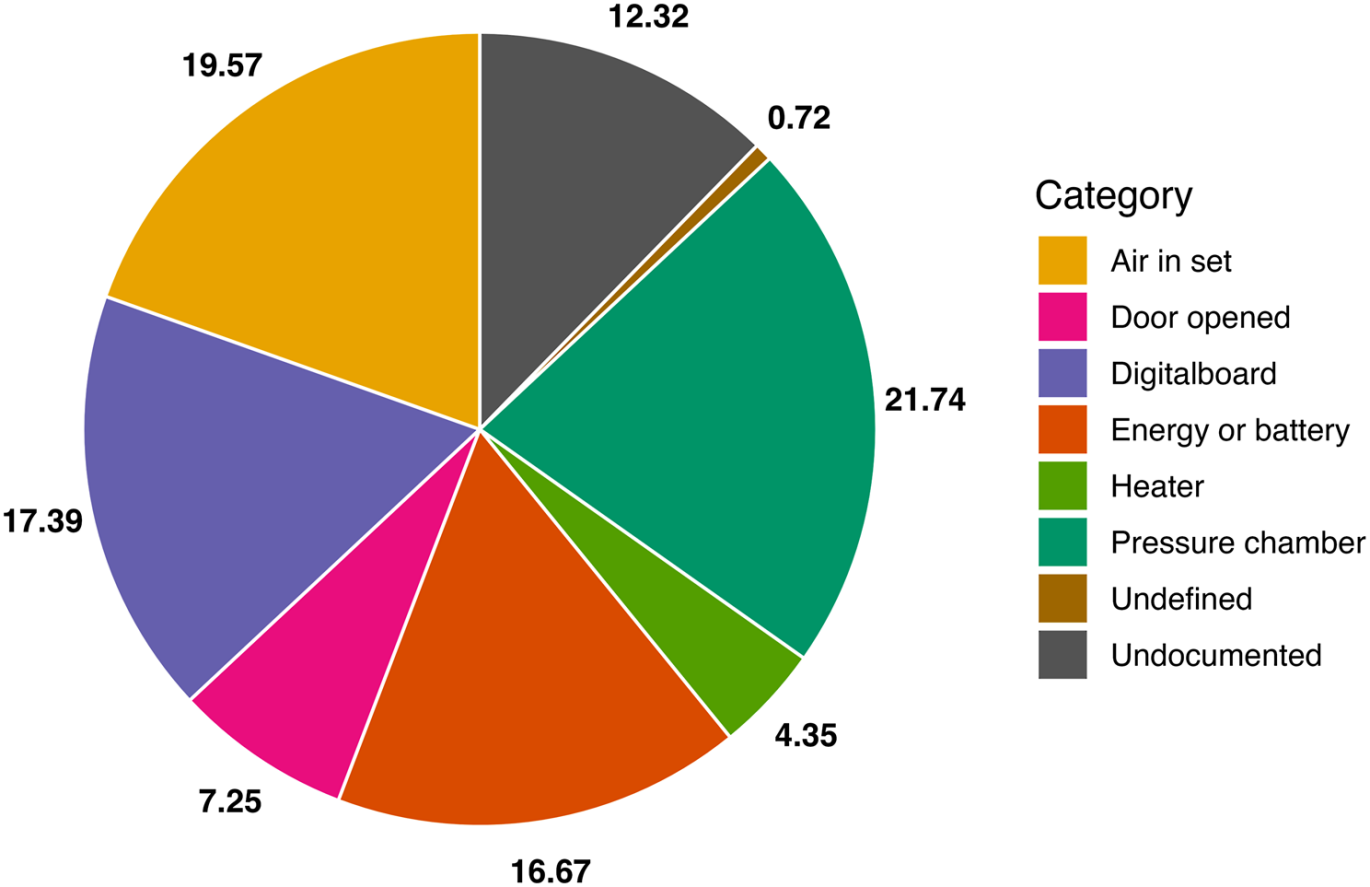
Pie chart illustrating the distribution of the total of *N* = 138 system errors in percent according to eight attributed categories

In the observational time frame including the years 2015–2023, in total 138 therapy interrupting system error incidents were registered. Those were assorted to 49 individual therapy interrupting system errors, characterised, and assorted to seven plus one (Undocumented) categories reflecting the attributed origin of issue (Fig. 1). Of those 48 could be described by their individual system error numbers, which consists of up to four digits (n–nnnn).

We categorized 18 out of 49 individual system errors to be associated to the ‘pressure chamber’ that in certain publications is also called the cassette chamber. Additionally, 18 errors were associated to the device’s Digitalboard/EEPROM (Electrically Erasable Programmable Read Only Memory). Three errors were attributed to the ‘detection of air in the disposable tubing set (air in set)’ and solution bag which may be associated to leaks, disconnected disposable tubing, loose connections, fluid level not at or near the patient connector after completing the prime cycle (incomplete priming) or unclamped, unused supply lines. Three errors were associated to heater malfunction, and three errors were associated to the ‘energy system or battery’, one of those without individual error number (readout as BATTERY ERROR). Another three errors were associated to door malfunctions such as insufficient or incorrect closure of the hatch or illegal opening during a running treatment program.

We categorized one system errors as ‘undefined’ since cause for the error message could not be determined by the patients or staff. Finally, error messages which patients or staff did not document the individual number for were labelled as ‘nnn?’ (Table 1).

### Frequency and characterisation of system errors

**Fig. 2:**
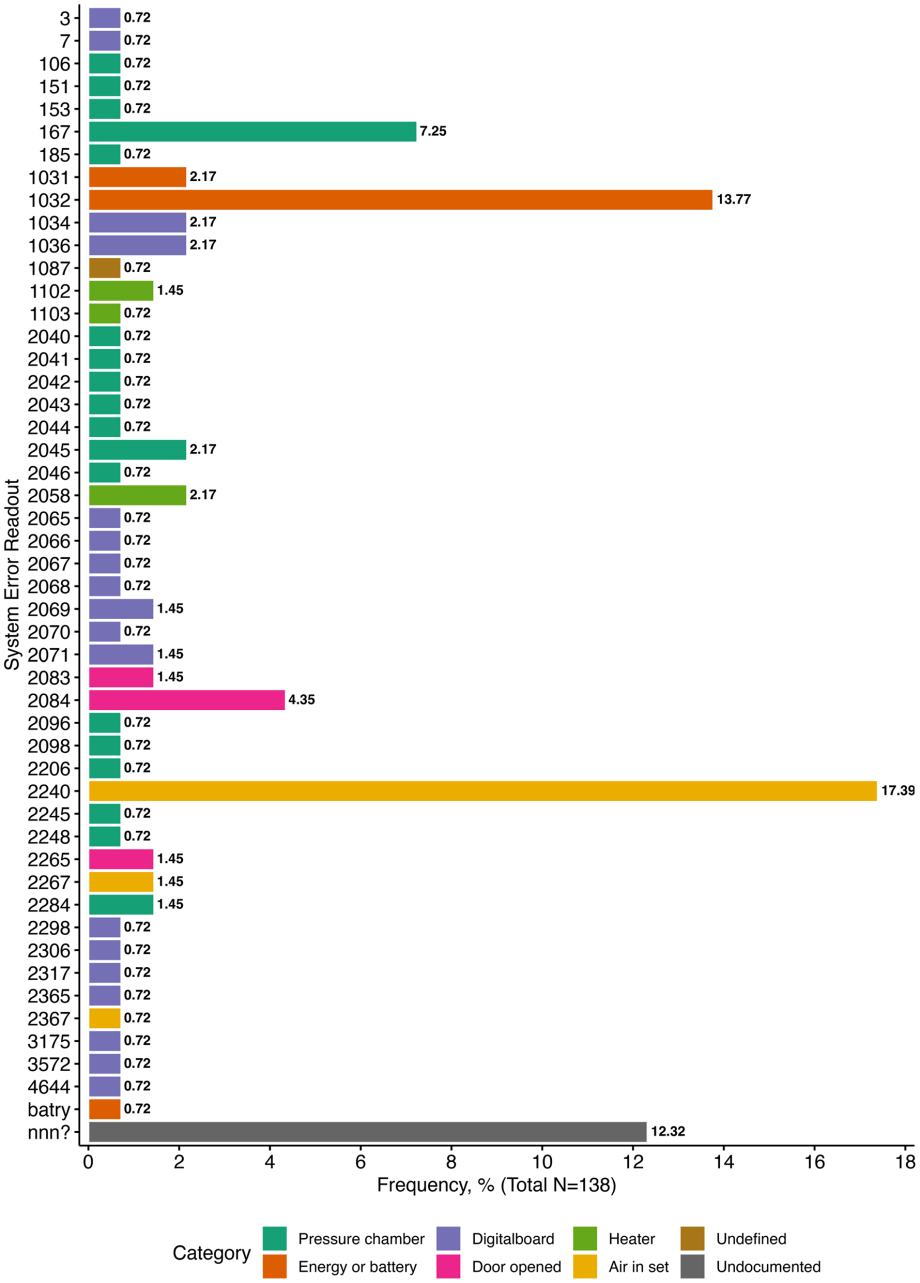
Bar chart illustrating the System error readouts assorted to eight categories. A total of 48 different system error number readouts were recorded. One error assorted to the energy or battery category did not identify by a specific number but the readout BATTERY ERROR. In a final category, error messages which patients or staff did not document the individual number for were labelled as ‘nnn?’. The most frequent errors were 2240 (air in set, 17.39%) followed by 1032 associated to the energy overload protection (13.77%). System errors related to the correct procession of dialysis fluid such as air in set or the cassette (19.57%) or correct closure of the door or hatch (7.25%) of/associated to the pressure chamber (21.74%) accounted for the majority of errors. Another three errors related to the power supply and accounted for 16.67% of total system errors that we observed. In 12.32% of errors that occurred during the observational timeframe a direct cause of the error could not be determined since the system error number readout was not documented

There were 18 individual errors associated to the Digitalboard/EEPROM or the pressure chamber accounting for 21.74% and 17.39%, respectively. In contrast, there were only three errors associated to the energy or battery category accounting for 16.67%. The single most frequent error 2240 attributed to air in set accounted for 17.39% of total errors, the second most frequent error 1032 associated to the energy overload protection accounted for 13.77% of total errors (Fig. 2).

The individual system error numbering indicates families and categories of specific system errors assigned to a sequence of numbers which represent related software- or technical functions (Table 1). For example, the system errors 2041–2046 are associated to the pressure chamber, while 2334–2389 point towards a series of problems with the Digitalboard/EEPROM. In our experience, three-digit system errors (1xx) were more frequently associated to internal part malfunctions and in many cases needed send-in service for part replacements.

System errors 1031–1032 are associated to an energy overload protection of the device. Switching off the cycler and disconnecting the power will enable successful start of the therapy after reinitiating the program.

In a minority of cases, switching off the cycler and reinitialising the program without change of the setup may enable the successful continuation of therapy. It is suggested to wait 30 s before switching on the device again. An in-depth characterisation of the recorded system errors and proposed resolution is summarized in Table 2.

## Discussion

In this retrospective study, we characterise and suggest resolutions strategies to approach therapy interrupting system errors that occurred during the use of APD HomeChoice cyclers. We identified previously unpublished system errors that were assigned to eight major categories: Errors readouts associated to the Digitalboard/EEPROM, the pressure chamber, air in the disposable tubing set, and those associated to the energy system or the heater resulted in abrupt termination or non-commencement of the treatment session without initial option to continue it. Most errors were attributed to the pressure chamber and the Digitalboard/EEPROM (sum 39.13%), while the most frequently occurring system error 2240 was associated to air the disposable set (17.39%) and the energy overload protection (1032, 13.77%).

In a prospective study on home haemodialysis, Reintjes et al. filed 172 incidents which most of (> 57%) were related to various technical aspects or machine setup troubleshooting [13]. We recently analysed the nature of emerging incidents from our PD patients directed at our on-call PD nurses during the after-hours [7, 14, 15]. A majority of problems reported from persons doing APD were related to technical problems. Hilbers et al. identified shortcomings in manufacturers’ technical documentation on the safe use of dialysis devices at home and emphasized the need for greater focus on user-related errors, home-specific design risks, and improved user information [16].

However, no previous study analysed therapy interrupting system errors of APD cyclers or provided applicable approaches to safely guide nurses and persons doing APD through the necessary error management.

As an advantage, specifically when using the HomeChoice cycler in the clinic, the accessible external set and lines approach make it possible to change the bag configuration during operation. However, special attention should be paid to breaking the seals of the connected bags. System errors regarding the pressure chamber are relevant as this component is responsible for the correct measurement of the patients’ fill volume. Interrupting the therapy in case of inconsistencies is necessary, since particularly infants and small children may be at risk in case of overfill [17]. In such cases it seems to be the best way to initiate a drain if already connected and subsequently replace the set to assure safe therapy. At this point it also should be stressed that sets, lines and bags should not be reused since contamination of the fluid or fluid pathways may result in peritonitis [18, 19].

When commencing the therapy system errors associated to air in set or the cassette system may be resolved by the option to vent the set or lines. However, this is only rarely possible because the cycler’s program sequences seem to be predetermined and usually do not offer any option for intervention for the person doing APD. Unforeseen interruptions in the cycling program do not seem to be embedded in the software such as an “what if … then”-option. In several cases, an experienced APD nurse may be able to vent the set and lines manually to address alarm messages, however, such a procedure cannot be recommended for laypeople and persons who have not been instructed in the medical-technical aspects of the device, and as such asepsis may get compromised. Additionally, only a limited number of alarms can be addressed by re-venting the set as air bubbles may not be sufficiently mobilised regardless, if venting is performed manually or using the menu system. Therefore, in most cases, the person doing APD has no other option but to change the set and replace the bag setup. Several system errors associated to air in set or the pressure chamber are preceded by alarms to check lines or bags that were potentially bypassed by the user without resolution. It is of relevance, that system errors such as 2240 occur as a safety aspect when there is more than approximately 3 mL of air in the system. Therefore, errors regarding air in the disposable set may likely be connected to errors associated to the pressure chamber: If there is air in the set or system, the pressure chamber may not be able to provide sufficient measurements regarding the flow volume. Insufficiently closing the pressure chamber’s door or hatch, manipulating, and twisting or warping the cassette ever so slightly during setup, may also lead to various associated error readouts indicating air trapping, leakage or door opening.

Errors may also relate to insufficient amount of dialysate in case of frequent cycle interruptions and very tight regimes when air is drawn from the empty bags instead. In these cases, we suggest withholding approximately 500 mL of the total dialysate for flushing of lines [7]. If venting attempts fail, and to ensure the adequate and safe course of treatment specifically regarding fill volume, we suggest to close the clamps, to switch off the cycler, replace the set and re-initialise the program. During cycles system errors will therefore frequently result in the need to early terminate the session. However, it is important to note, that nearly half of all errors registered were associated to air in set, the pressure chamber or its door or hatch and may be likely caused by user handling error during system setup. If these occur frequently, we suggest retraining of the user.

A Baxter publication hosted by the German Federal Institute for Pharmaceuticals and Medical Products, informs users of the HomeChoice PRO cycler about several instructions for safe operation of the device [20]. The information concerns the problems of initial drain alarm, system errors as well as abort and disconnection measures. Additionally, the manual of Baxters’ latest cycler the HomeChoice Claria (manual ver. 07-19-65-355B1 and older versions for the PRO cycler, 07-19-61-244) feature the troubleshooting and most frequent triggers of system errors 2240 and 2267. The follow up error 2367 is also addressed. Baxter specifically advises that in case of a system error the user should not open the door to the pressure chamber until all clamps are closed to prevent gravity flow of fluid from one bag to another, and/or to the patient. Uncontrolled gravity flow of fluid can result in an intraperitoneal volume overfill situation or potential risk of pneumoperitoneum in case of air insufflation [21].

Further in detail descriptions of error messages specifically those regarding the Digitalboard/EEPROM, which is a functional system memory are not released as these are primarily aimed at service technicians. Unfortunately, system errors may lead to a loss of responsiveness of the device menu. It is then no longer possible to override the current program to initiate a drain and subsequently activate the last bag fill cycle. The person doing APD must then perform a gravity drain and fill in the last bag manually. In cases of non-responsiveness, switching off or disconnecting the cycler from the power supply may empty the cache and re-initialising the therapy may be possible in some cases. This is due to safety reasons, as during the first 30 min of a power failure, the cycler will be able to retrieve the ongoing therapy information. Within two hours the cycler may also be able to continue the program. Restarting after replugging the system to the power supply with setup completed also works for errors associated to the energy overload protection of the device.

In our centre we provide more than 7.500 patient days of APD treatment per year (mean). In general, we feel that therapy interrupting system errors on the HomeChoice system are rare (< 0.25% of sessions performed). Still, our findings may have several important implications for staff and patients alike. Teaching patients to find and address the exact problem for each alarm remains an ongoing and unpleasant process for nurses [22]. For example, during treatment, the cycler will no longer differentiate between the direct source of obstacle in the setup and any insufficient venting caused by a bags’ mis- or disconnection may be a cause of program interruption [7] with a potential readout of system error 2240. For users who are rarely faced with such problems, tackling of errors must be unsettling [23]. However, information presented in this manuscript may be useful to inform staff and train patients on error-prone details when setting up the system. We suggest that improvements delivering more patient-friendly display with simple guidance on errors might be helpful to improve patient experience such as the information to follow the given instructions from the APD device in case of system error (e.g. “restart the system after reconnection of power supply”, “change set and bags and restart the program”) [24]. With the Claria the Sharesource connectivity provides troubleshooting information and error readout as well as options for online adaption of therapy which benefits patients to have better sleep and improved confidence in their therapy [25]. Accordingly, the Sharesource platform could be expanded with more patient friendly troubleshooting information, error readout and options for intervention in therapy interrupting system errors by the staff. At present those data sent to the cloud can only be accessed some time after cycler switch off or successful finishing of a therapy session.

Accordingly, in cases of system errors interrupting the prescribed therapy program during nighttime, our centre’s patients are instructed to either switch off the cycler and disconnect in the morning or safely disconnect if they feel confident about it, rather than changing bags, reconnect lines or bags to the cycler in ongoing treatments, as this may compromise setup integrity and asepsis [26]. Some patient groups receiving APD, such as infants, children, as well as an increasing number of patients with cardiorenal syndromes and chronic heart failure doing APD, are more susceptible to the adverse effects of fluid retention [27, 28], that may be caused by interrupting a prescribed therapy. Any discontinuation may therefore be of greater importance than for those with incremental PD prescriptions and less compromised diuresis.

This observational study was part of a quality improvement program, using routinely collected health data from medical records of a representative outpatient PD-cohort. As a limitation, we did not systematically document data on therapy continuity in cases of errors during regular office hours and not all incidents may have been documented with similar detail. External validity and representativeness may therefore be limited, and the information provided in the present manuscript may not universally apply to other cycler systems such as the Amia that currently is not available in Europe. Additionally, this curation of errors and user experience in our center makes no claim to provide a complete listing of potential system errors. Categories, issue and aggregated resolving solutions hence should not be considered as clinical guideline or medical advice and some of the system errors listed here may have already been resolved by manufacturers’ updates.

There is continued interest to increase the number of patients treated with home dialysis options [29, 30]. Therefore, more and more patients will be tasked/confronted with performing these complex and technical medical procedures at home. However, especially in the older generation, independent use of technology and software cannot be expected, and simplification of the technology and software of APD-systems is needed to improve their therapy experience [31]. Especially for the persons using APD cyclers daily, future developments in home monitoring and telemedicine may facilitate these procedures and further improve quality of life and support options [32, 33].

## Conclusion

Therapy interrupting system errors on the HomeChoice systems are frequently associated to the Digitalboard/EEPROM, the pressure chamber or insufficient venting of the set and lines, which may be caused by handling errors during setup. As safety precautions a relevant number of system errors will unequivocally terminate the treatment session with the need to replace the setup and reinitialise the program. In our cohort, however, such errors appear to be rare regarding the total number of treatments performed. Our findings may be useful for training and educational purposes for nephrology personnel. For the manufacturer, the information provided may be helpful to improve error management and information transmission to the user.

## Data Availability

The datasets generated and/or analysed during the current study are not publicly available due to restrictions specified in the study protocol but are available from the corresponding author on reasonable request.

## Abbreviations

APD: Automated peritoneal dialysis
EEPROM: Electrically Erasable Programmable Read Only Memory
PD: Peritoneal dialysis
